# Recent advances of nanocrystals in cancer theranostics

**DOI:** 10.1039/d3na00397c

**Published:** 2023-07-10

**Authors:** Devyani Yenurkar, Malay Nayak, Sudip Mukherjee

**Affiliations:** a School of Biomedical Engineering, Indian Institute of Technology, BHU Varanasi-221005 UP India sudip.bme@iitbhu.ac.in

## Abstract

Emerging cancer cases across the globe and treating them with conventional therapies with multiple limitations have been challenging for decades. Novel drug delivery systems and alternative theranostics are required for efficient detection and treatment. Nanocrystals (NCs) have been established as a significant cancer diagnosis and therapeutic tool due to their ability to deliver poorly water-soluble drugs with sustained release, low toxicity, and flexibility in the route of administration, long-term sustainable drug release, and noncomplicated excretion. This review summarizes several therapies of NCs, including anticancer, immunotherapy, radiotherapy, biotheranostics, targeted therapy, photothermal, and photodynamic. Further, different imaging and diagnostics using NCs are mentioned, including imaging, diagnosis through magnetic resonance imaging (MRI), computed tomography (CT), biosensing, and luminescence. In addition, the limitations and potential solutions of NCs in the field of cancer theranostics are discussed. Preclinical and clinical data depicting the importance of NCs in the spotlight of cancer, its current status, future aspects, and challenges are covered in detail.

## Abstract 2

This review summarizes the examples of cancer theranostic applications of different NCs.

## Graphical abstract

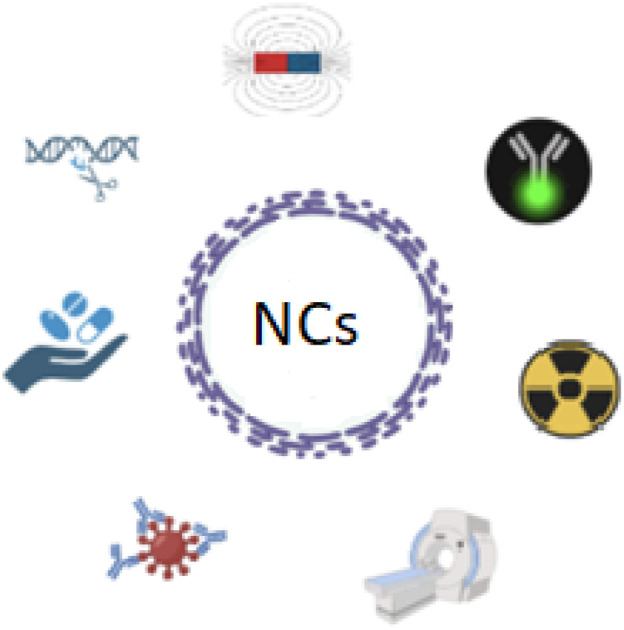

## 1. Introduction

Cancer is a complicated illness with nearly 200 forms and is a significant global burden.^1^ Cancer cells grow uncontrollably, invading the surrounding tissue, resulting in localized injury, inflammation, and metastasis. Many factors contribute to cancer development, characterized by abnormal growth of human cells, including environmental pollution, genetic changes, food and lifestyle changes, and virus and microorganism infections.^2^ More than 10 million people died from cancer globally in 2021, emphasizing one in every six fatalities.^3^ Cancer still poses a challenge to world healthcare despite significant advances made over the past 40 years to improve its detection and treatment.^4^ Statistics from the American Cancer Society indicate that by 2030, there will be 21.7 million additional instances of cancer worldwide. The market for cancer pharmaceuticals was estimated to be worth $135494.17 million in 2020, and it is anticipated to grow to $274400.63 million by 2030, representing a 7.5% CAGR between 2021 and 2030.^5^ The conventional therapies include surgery, radiation therapy at the initial stages, and chemotherapy in later stages or a combination therapy. Other recent therapies include photodynamic therapy,^6^ gene therapy,^7^ immunotherapy,^8^ cell therapy,^9^ therapeutic vaccines,^10^ and antibody therapy,^11^ or as a combination of these.^12^ Limitations, including long-term toxicities, acquired drug resistance, lack of targeting, and poor drug bioavailability, cause conventional therapies to be ineffective.^13,14^ Hence, finding a cost-effective alternative therapy with better targeting, increased bioavailability, and reduced side effects is urgently required.^15^ In this context, nanotechnology can significantly contribute to overcoming the drawbacks of the traditional therapeutic approach. Novel drug delivery systems (NDDS) such as nanocarriers (nanoparticles and nanocrystals) are emerging for the development of alternative theranostics due to their distinct fundamental qualities (high surface energy, high surface-to-volume ratio, and small size) compared to bulk materials, improvement of drug solubility and bioavailability, reduction of drug dose, and curbing down side-effects.^12,16–20^

Nanocrystals are carrier-free solid drug particles that are sized in the nanometer range containing distinguished crystalline characteristics. Nanocrystals have a special role in nanotechnology-based strategies for targeting tumors as they are accessible for active and passive targeting with maximum drug content and increased bioavailability for poorly soluble drugs.^21,22^ In general, NCs show higher size than NPs. Moreover, organic nanosized crystals are more common than others and can be used for drug delivery applications without any delivery vehicle. Moreover, due to extraordinary drug loading (∼100%) and the minimal presence of organic solvents or solubilizing chemicals, NCs have appealed enormous interest in the field of drug delivery treating different cancers. Compared to nanoparticles (NPs), NCs have certain benefits because of their distinct electrical and optical characteristics from quantum confinement effects.^23^ The efficiency of their photoluminescence is likewise quite high compared to NPs, and they produce light with a limited and well-defined spectrum of wavelengths and little energy loss. Due to their effective emission, they are helpful in applications such as lighting, displays, and biological imaging.^24^ NCs are resistant to degradation in terms of stability and durability.^25^ One specific advantage over NPs is that NCs can be implantable.^26^ NCs are solid nanosized (10–1000 nm) crystalline particles, which enhance the bioavailability and increase the solubility because they are recognized as colloidal dispersion consisting of 100% active pharmaceutical ingredients (API) without any additional materials such as lipids and polymers.^23,27,28^ NCs occur in various forms, including organic,^29^ inorganic,^30^ and hybrid NCs,^31^ each having distinct features and applications. Organic NCs involve preparation through organic materials such as cellulose. They are little challenging in the synthesis as they possess poor thermal and mechanical properties.^32^ Inorganic NCs comprise semiconductors, metal, and magnetic-based NCs. They are more stable, biocompatible, nontoxic, and hydrophilic than organic NCs.^33^ The term hybrid indicates blending different materials with unique properties to create a new material with enhanced or synergistic characteristics. By combining the properties of different materials, hybrid nanocrystals can exhibit improved performance in cancer theranostics.^34^ In this review article, we summarize the cancer theranostic applications of NCs and highlighted the various drawbacks and potential solutions. Moreover, the preclinical and clinical status of these agents are reported.

## 2. Types of nanocrystals

### 2.1. Organic nanocrystal

**Fig. 1:**
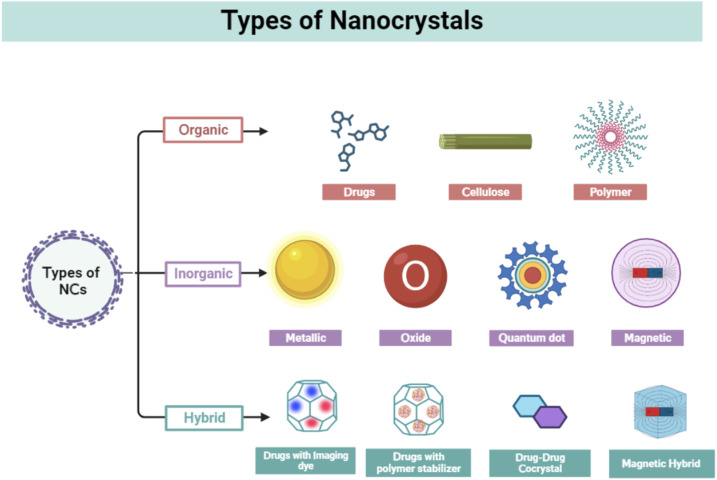
Types of nanocrystals; figure is created with https://www.BioRender.com.

Organic NCs are tiny structures made from organic molecules such as cellulose,^35^ drugs (*e.g.*, cabazitaxel, resveratrol, and paclitaxel) polymers, and hemoglobin.^29^ One of the commonly used methods for the synthesis of organic NCs is mechanical milling.^36^ These are frequently produced by condensation and precipitation processes that use synthetic methods or self-assembly principles employing bottom-up techniques.^37^ Other synthesis methods include laser ablation,^38^ solvent-vapor annealing,^39^ sonication technique,^40^ and sol–gel coatings. Organic NCs have a general size of more than 100 nm that can reach up to 1 μm. The organic molecules play an important role to modulate the size and geometry of the synthesized NCs. Various strategies are frequently used to create homogeneous cellulose NCs including acid hydrolysis, enzyme-assisted hydrolysis, and oxidation by disrupting the hydrogen bonding network and breaking the cellulose microfiber.^41,42^ In one of the investigations, cellulose NCs were formed by attaching a carboxylic group with presynthesized *cis*-aconite-doxorubicin by the amide linkage used for anticancer drug treatment.^43^ Organic drugs and molecules such as 10-hydroxycamptothecin,^44^ oridinon, ursolic acid (UA),^45^ paclitaxel,^46,47^ and silybin^48^ have been in use for cancer treatments, in the form of NCs by the improvement of bioavailabilities and the reduction of toxicities of these drugs. In 2023, Zhou *et al.* synthesized hemoglobin NCs through the batch crystallization method and utilized it for colon tumor-specific diagnostics and therapeutics.^29^ Overall, biological and organic NCs such as hemoglobin NCs (HbC) offer advantages such as low toxicity, the ability to target cells, excellent tissue penetration, biodegradability, and simplicity of manipulation. On the other hand, large size, tissue accumulation, allergic responses, and efficacy are the primary drawbacks of organic NCs (Fig. 1).^49^

### 2.2. Inorganic nanocrystals

Inorganic compounds such as metals, semiconductors/metalloids, metal oxides, magnetic materials, and ceramics are involved in the synthesis of NCs. These have sizes ranging from 1 to 100 nm. Because of their distinctive optical, magnetic, and fluorescent features, these NCs have received much attention for therapeutic and diagnostic uses. They have been differentiated based on their nature and properties. Quantum dots are composed of semiconductor materials and exhibit size-dependent optical properties such as tunable absorption and emission wavelengths. This makes them valuable for displays, lighting, solar cells, biological imaging, and sensing applications.^50^ Using PEG, imidazole, and phosphoric acid, V. Bajpai *et al.* created nitrogen phosphorus-doped quantum carbon dots for anticancer properties. These carbon quantum dots exhibit apoptotic properties, with cell cycle arrest and autophagy, resulting in cell death in B16F10 melanoma cancer cells.^30^ Metal NCs can be formed from various metals, such as gold, silver, platinum, or copper. They exhibit localized surface plasmon resonance (LSPR), which leads to enhanced light absorption and scattering. They have applications in catalysis, electronics, photonics, and biomedical sensing.^51^ Palladium NCs coated with gold were created through the seed growth method by A. McGrath *et al.* They show successful photothermal applications by killing HeLa cells *in vitro* and destroying cancer tissues HeLa tumor model *in vivo*.^52^ Magnetic NCs are composed of iron, gadolinium, or cobalt (Co) oxides. They exhibit ferromagnetic or superparamagnetic behavior, depending on their size, and are used for data storage, magnetic resonance imaging (MRI), drug delivery, and magnetic hyperthermia in cancer theranostics.^53^ One such example is superparamagnetic iron oxide (Fe_2_O_3_ and Fe_3_O_4_). For a range of biomedical uses involving contrast agents for MRI, therapeutic gene carriers, protein extraction, and nucleic acid and virus detection, iron oxide (IO) nanocrystals have drawn considerable interest. In an article published by Y. Lee *et al.*, IONCs with hyaluronic acid coating were synthesized from the thermal decomposition method for targeted cancer imaging.^54^ In another study, coprecipitation and partial oxidation technique were used for the synthesis of IO NCs for the application of MR and NIR absorption imaging.^55^ Inorganic NCs can be easily modulated to any size and shape. Due to their smaller size compared to organic NCs, inorganic NCs demonstrate higher more surface area and drug loading capacity. Moreover, it lacks biocompatibility and shows higher cytotoxicity both in cells (*in vitro*) and in animal models (*in vivo*), making them unsuitable for cancer theranostics.^49^

### 2.3. Hybrid nanocrystals

Hybrid NCs have received much interest due to their remarkable imaging property where the drug crystals behave as hosts and form composites with molecules of interest. The concept is based on a well-known occurrence in solid-state chemistry when impurities are incorporated into a host's crystal structure, altering their optical, mechanical, and electronic properties.^56^ However, integrated dye molecules produce little effect on drug nanocrystals' crystallinity and biological functional properties. Over a decade, scientists have prepared several hybrid NCs using various drugs such as lapatinib, camptothecin, paclitaxel, quercetin, itraconazole, saquinavir, and cyclosporine. Rhodamine, Cy5, gold, DiD, DiR, FPI-749, FPR-749, FPR-648, and fluorescein were used as dopants to the host particles for the application of cancer imaging. C. P. Hollis *et al.* reported the synthesis of organic camptothecin-doped Au NCs by incorporating Au in the host crystal. Here, camptothecin helps in killing colon cancer cells, whereas gold acts as an image contrast agent for bioimaging.^31^ In another study, hydroxyapatite NCs were prepared with tris(2,2,6,6-tetramethyle-3,5-heptanedionato)europium(iii) (EuTH) complex, developing a luminescent organic–inorganic hybrid NCs.^57^ Further, these NCs were conjugated with folic acid derivatives for targeting HeLa cancer cells (Table 1).

**Table 1:** Examples of some recently developed nanocrystals used in cancer theranostics

Serial no.	Nanocrystal	Type	Preparation technique	Size	Function	Ref.
1	Resveratrol NCs	Organic	Wet media milling	270 ± 7.2 nm	Anticancer agent	58
2	Haemoglobin NCs (hbc)	Organic	Batch crystallization	370 nm	Synergistic theranostics of colon tumor	29
3	Cellulose nanocrystal (CNC)	Organic	Acid hydrolysis	193 nm	Enhanced retention in pulmonary metastasis of melanoma	59 and 60
4	Cellulose NCs loaded with 5-fluorouracil	Organic	Coprecipitation method	87.12 ± 5.6 nm	Colorectal cancer treatment	35
5	Salinomycin NCs	Organic	Antisolvent, bottom-up precipitation augmented by probe sonication	210 ± 10 nm	Colorectal cancer treatment through inhibition of Wnt/β-catenin signaling	61
6	Folic acid and poly(ethylene glycol) decorated paclitaxel NCs	Organic	Bottom-up method	201.90 ± 2.92 nm	Breast cancer-targeting	62
7	Albumin-coated carfilzomib NCs	Organic	Crystallization in surfactant-containing medium, followed by surface coating with albumin	270.8 ± 21.5 nm	Breast cancer therapy	63
8	Cabazitaxel (cbtx) NCs	Organic	Bottom up	108 nm	Targeted therapy for metastatic breast cancer	64
9	Ultrasmall γ-Fe_2_O_3_ NCs	Inorganic	One-pot hydrothermal strategy	280 nm	MRI imaging and photothermal therapy	65
10	Hexagonal NCs of Na_*x*_WO_3_	Inorganic	Hydrothermal method	100–150 nm	Thermal and chemodynamic combined cancer therapy	66
11	Hafnium oxide NCs	Inorganic	Microwave-hydrothermal (MH) method	65 nm	Enhanced radiotherapy efficacy for cancer theranostic	67
12	Mn-doped ZnO fluorescent NCs	Inorganic	Chemical hydrolysis method	—	Cancer theranostics (imaging)	68
13	Iron-doped palladium NCs	Doped inorganic	Thermal decomposition	52.66 nm	Enhanced hydroxyl radical generation for chemo-/chemodynamic nanotherapy	69
14	Silica-coated Mn-doped ZnS NCs	Doped inorganic	Vapor diffusion	>200 nm	Cancer theranostics	70
15	GdOF NCs	Inorganic	Homogeneous coprecipitation method and high temperature (500 °C) calcination	500 nm	Cancer theranostics	71
16	Near-infrared iron oxide NCs	Inorganic	Ligand-assisted coprecipitation method	150 nm	Magnetically targeted imaging and photothermal cancer therapy	55
17	Magnetic CuFeSe_2_ ternary NCs	Inorganic	Wet chemistry method	4.1 ± 0.4 nm	Multimodal imaging guided photothermal therapy	72
18	Paclitaxel-loaded magnetic NCs	Hybrid	High-temperature pyrolysis	166.4 nm	Synergistic therapy combining magnetic hyperthermia with chemotherapy	73
19	Europium(iii) complex-based hydroxyapatite NCs	Hybrid	Precipitation	49 nm long	HeLa cancer cell imaging	57
20	Platelet membrane-cloaked pegylated-paclitaxel NCs	Hybrid	Modified emulsion-lyophilized crystallization	432.97 ± 51.94 nm	Postoperative chemotherapeutical efficacy	74

## 3. Nanocrystals application in cancer therapy

**Fig. 2:**
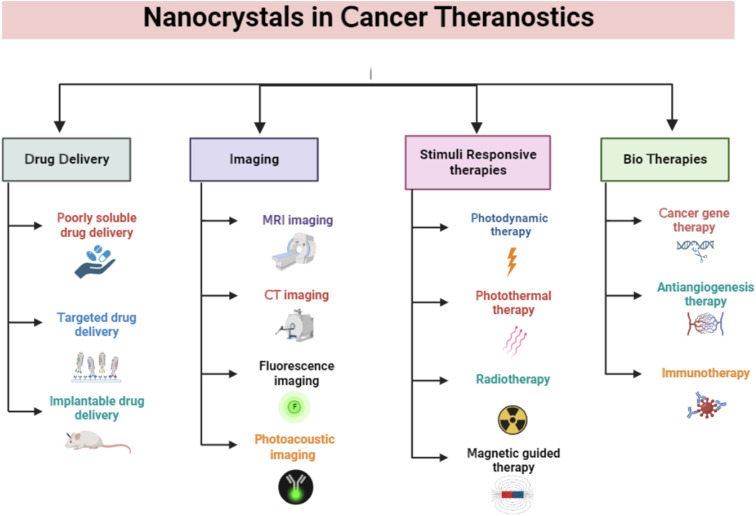
Applications of nanocrystals in cancer theranostics. The figure is created with https://www.BioRender.com.

Nanocrystals have drawn much interest in cancer therapy due to their ability to deliver poorly soluble drugs, high loading efficiency, improved stability, and the prolonged release of drugs. NCs have several different uses in cancer theranostics, including drug delivery, anticancer therapy, immunotherapy, biotherapeutics, radiotherapy, photothermal therapy, imaging, and biosensing (Fig. 2).

### 3.1. Nanocrystals as drug delivery vehicles

NCs are used to develop novel drug delivery systems by incorporating anticancer drugs inside them for delivery to the desired site as an implantable form. Poorly soluble drug NCs are further encapsulated using a suitable biomaterial for developing an implantable therapy.^75^ Physicochemical properties, such as surface modification and size distribution, enable the NCs to be used in drug delivery, especially for cancer treatment.^76^ To evaluate the anticancer efficacy for colorectal cancer utilizing an *in vitro* tumor-on-chip model, M. Yusefi *et al.* synthesized cellulose NCs (CNCs) loaded with fluorouracil. The CNCs were rod-shaped and extracted from rice straw waste. When fluorouracil was used in CNCs, it was shown to have improved thermal stability. In addition, at pH 7.4, the drug release was better. The high biocompatibility of CNCs was observed in normal colon cells, whereas it increased the anticancer effects of fluorouracil when treating various colorectal cancer cell lines (HCT116 and HT-29). Mechanistic studies showed that the stimulation of cell apoptosis and disruption of the mitochondrial membrane is the main reason for the anticancer activity.^35^ T. Li *et al.* discussed the significance of the shape and size of NCs for drug release. In this work, liposomal ciprofloxacin NCs were produced by altering the arrangements of the lipid bilayer.^77^ This was accomplished using several membrane phospholipids with variable cholesterol content ratios. Phospholipids directly played an important role in the modulation of NC size. Adding cholesterol decreases the fluidity and permeability of liposomes. A higher drug loading can result in sustainable drug release, resulting in improved therapeutic efficiency and lower toxicity.^77^ In another study, Fe_3_O_4_ NCs containing curcumin were produced and demonstrated biocompatibility when tested in brine shrimp.^78^ In addition, these NCs offered magnetically-induced accelerated drug release influenced by external magnetic fields. These NCs stimulate an apoptosis-mediated reduction of colon cancer cells, as confirmed by the MTT assay. Using an HCT116 3D colorectal tumor model, it was shown that the NCs were able to reduce the tumor volume twice in comparison to the control group. Due to its enhanced drug delivery, it may be regarded as the most effective yet promising drug delivery method for the magnetic-triggered release of bioactive treatments.^78^

**Fig. 3:**
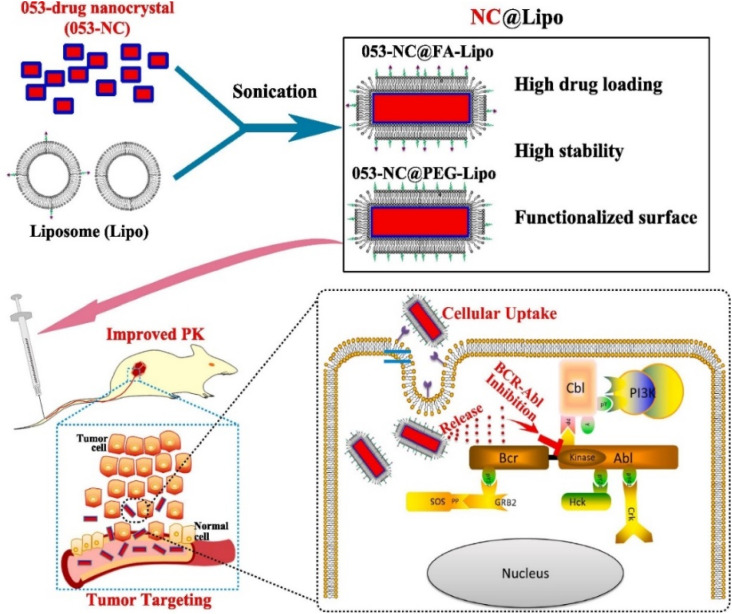
Fabrication of NCs to target tumor cells after intravenous injection into the mice model. Reprinted with permission from ref. 79. Copyright 2022, *Pharmaceutics*.

In a research work (Fig. 3), H. Liang *et al.* developed a liposome-encapsulated with a hydrophobic drug (CHMFL-ABL-053) NCs (NC@Lipo), delivering them to the specific tumor location with high loading efficacy.^79^ CHMFL-ABL-053 is a selective BCR-ABL inhibitor for chronic myeloid leukemia, demonstrate poor water solubility, and reduced bioavailability. The formation of NCs helps in long-lasting drug release. Further, the liposome helps in controlled drug release, higher biocompatibility, and improved colloidal stability. Folic acid was further used to functionalize the NCs' surface for improved cancer targeting. PEG was attached to provide extended stability. The K562 xenograft mice were used to demonstrate the *in vivo* anticancer effects of the NCs with enhanced tumor accumulation. In peroral administration, the tumor growth inhibition rate was 48.3% at 50 mg kg^−1^. The longer circulation of drugs results in improved tumor growth inhibition.^79^ D. Zhao *et al.* prepared an injectable paclitaxel (PTX) and niclosamide (NLM) NCs coloaded with thermosensitive hydrogel for triple-negative breast cancer treatment.^80^ PTX and NLM NCs were prepared by 3PNET and antisolvent precipitation methods, respectively. These exhibited sustained drug release from gel for eight days *in vitro*. It displayed a synergistic effect by blocking the migration and proliferation of cells along with apoptosis. *In vivo* studies were carried out in female BALB/c nude mice for 20 days in the breast cancer tumor model. This gel suppressed breast cancer stem cells (BCSCs) when injected, leading to effective breast cancer treatment.^80^ By creating NCs of the pure drug SN-38 *via* the ultrasound-assisted reprecipitation method, Y. Koseki *et al.* demonstrated effective drug delivery.^81^ Nanofibers and NCs of 150 nm in the shape of rods were produced. Higher drug loading can be effective for delivering drugs through NCs. These drug NCs demonstrated better therapeutic efficacy compared to the SN-38 prodrug.^81^ In another study, Z. Luo *et al.* created prefabricated cabazitaxel NCs to target the spread of metastatic progression *in vivo*.^64^ An orthotopic breast cancer model and a brain metastatic breast cancer model were used for evaluating the efficacy of the cabazitaxel NCs. These NCs use functional plasma proteins to bind to primary tumors, CTCs, and metastatic tumors, causing ligand-mediated targeting *in vivo*.^64^

#### 3.1.1. Nanocrystals in targeted therapy

In targeted drug delivery, a significant amount of the drug is accumulated in a region that is being treated. The targeted administration of drugs using NCs uncovers a particular interaction with a receptor in the targeted tissues.^82^ To improve the pace at which tumors are suppressed and thus produce a greater therapeutic impact, passive targeting can effectively increase the accumulation of drugs in the tumor tissues. It can also be paired with active targeting to concentrate drugs in the tumor tissue, which are subsequently taken up by tumor cells to produce apoptosis.^83^ Active targeting is meant to allow the drug into the tissue at the molecular level.^84^ In targeting based on the receptor, on the surface of tumor cells, overexpressed receptors may precisely identify their respective ligands and then take up nanocrystals into ligand–receptor-induced endocytosis of tumor cells for treatment.^85^

**Fig. 4:**
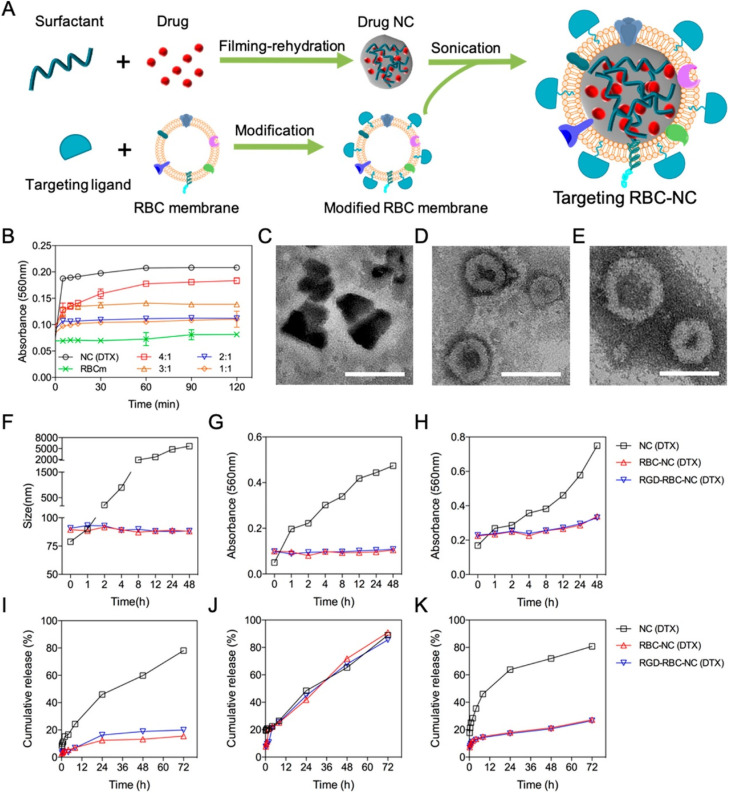
(A) Preparation of targeted RBC-NC. (B) Short-term serum stability study. (C–E) TEM images. (F–H) Long-term stability study of different DTX formulations. (I–K) *In vitro* drug release from different DTX formulations. Reprinted with permission from ref. 86. Copyright 2019, *ACS Nano*.

In a study (Fig. 4) coating the cell membrane, Z. Chai *et al.* reported targeted drug delivery through NCs.^86^ The procedure of filming-rehydration with F127 as excipient was used to prepare the NCs. The interactions between avidin and biotin allowed for the simple insertion of targeting peptides into the red blood cell (RBC) membrane. Prolonged stability and retention time, along with high drug loading and biocompatibility, were observed with the drug NCs coating on the RBC membrane (RBC-NCs). Fig. 4B explains the optimization of DTX mass to RBC membrane ratio by a short-term serum stability study. To create NC(DTX) and RBC-NC(DTX), we utilized the toxic insoluble chemotherapy drug docetaxel (DTX), which has a high molecular weight. Using a lipid insertion method, streptavidin was inserted into the RBC membrane.^86^ Biotin-linked c(RGDyK) was subsequently added and redesigned along with avidin and biotin reactions. The fusion of the RBC membrane with the NC(DTX) has been verified by TEM analysis. The irregular shape of NC(DTX) and the average size of 70 nm for RBC-NC(DTX) and RGD-RBC-NC(DTX) were identified; Fig. 4C–E shows the TEM images. *In vivo* safety research demonstrated the lower systemic toxicity of RBC-NC(DTX) and RGD-RBC-NC-(DTX) compared with NC(DTX). Fig. 4F–H shows long-term stability studies. *In vitro* investigations confirmed sustained phosphate-buffered saline (PBS) and serum durability. Fig. 4I–K represents the *in vitro* studies of formulations. U87 cells were utilized for cell culture.^86^ The antiorthotropic glioma and antisubcutaneous tumor models improve the treatment effectiveness, and more drug accumulation was observed at the tumor site, thus enhancing the anticancer activity. It can be used for targeted drug delivery as NCs gained active targeting capability through RGD modification.^86^ S. Wu *et al.* created liposomes containing cabazitaxel (CBZ) NCs for glioma-targeted treatment.^87^ These liposomes were modified by changing the geometry of the targeted ligand. The mouse orthotopic glioma model served as the *in vivo* testing platform. PV-Lip/cNC therapy accounts for the increased accumulation of CBZ in both the antiglioma and glioma impact. These NCs had a longer survival period (53 days). The findings showed that lipid NCs could cross the blood–brain barrier (BBB) and the blood–brain tumor barrier (BBTB).^87^ In addition, they can eliminate vasculogenic mimicry (VM), which will kill glioma cells for treatment. They found that the core of pure drug NCs allowed for high drug loading, whereas lipid membrane coating appeared to be a viable strategy to speed up circulation and stability. They also demonstrated outstanding results in penetrating tumor spheroid *in vitro* and passing the barriers and homing gliomas.^87^ The NCs can predominantly target the tumor cells and provide specific action on specific sites. This can be advantageous for using more such formulations targeting the desired tumor site rather than proving nonspecific action, leading to side effects.

### 3.2. Nanocrystals as anticancer agents

Drug resistance is the leading cause of cancer therapy failure.^88^ NCs as anticancer agents are superior to matrix-based nanomedicines with their high drug loading and active targeting, thus accelerating the clinical translation process in cancer therapy.^89^ NCs possessing prolonged and controlled drug release, efficient tissue penetration, enhanced cancer cell uptake, and improved organelle-targeted delivery can maximize cancer targeting.^90^ To treat colorectal cancer, Z. Wang *et al.* developed salinomycin NCs using a bottom-up antisolvent precipitation approach.^61^ This occurs by limiting and boosting the Wnt/β-catenin pathway's inhibitory effect. These NCs showed improved size distribution, stability, and water solubility. Fluorescence imaging investigations also explained the improved *in vivo* cellular uptake efficiency. These NCs showed significant 1.5–3 times increased cytotoxicity and inhibitory effect ten times better than that of pure drugs.^61^ For *in vivo* antitumor efficacy, transgenic mice were employed. When administered orally, the anticolon tumor effects in NCs were two-fold better than that of pure drugs. The research results indicated that greater tumor tissue accumulation and cellular internalization might represent a key strategy for treating colorectal cancer.^61^

**Fig. 5:**
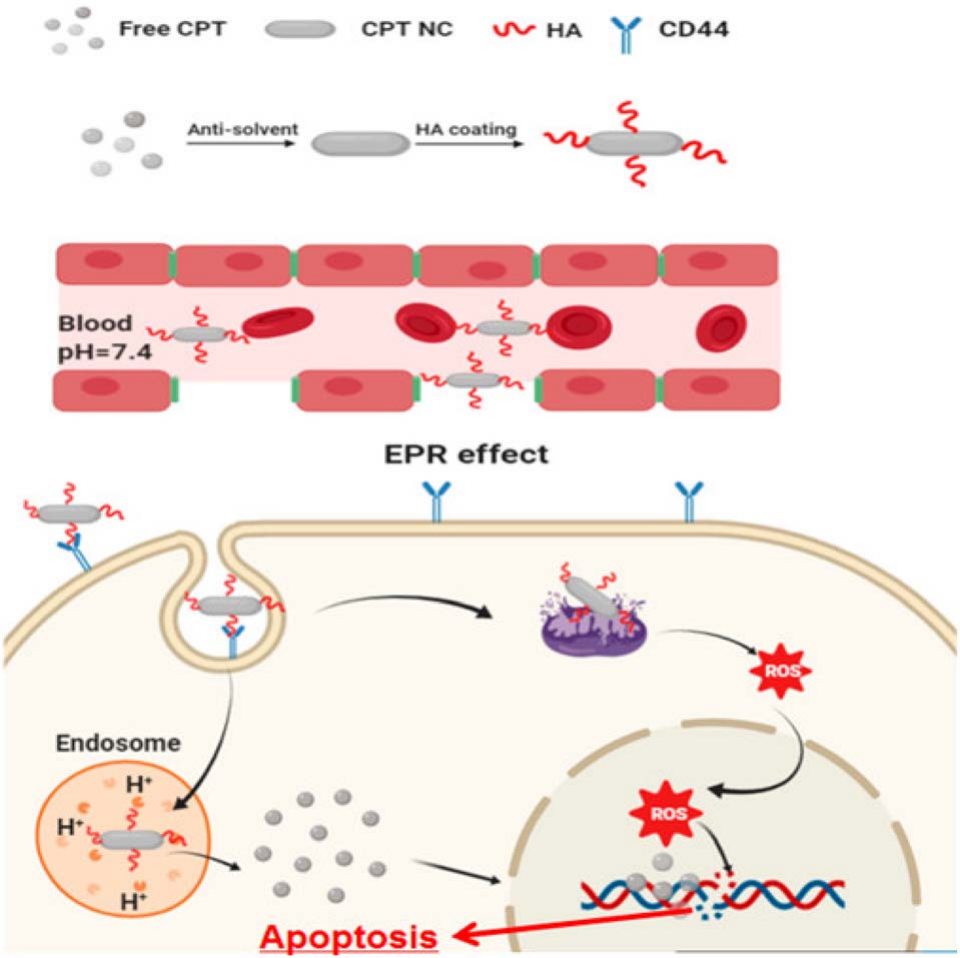
Hyaluronic acid-coated camptothecin (CPT) NCs with its mechanism to treat cancer. Reprinted with permission from ref. 91. Copyright 2020, *Molecular Pharmaceutics*.

Hyaluronic acid-coated camptothecin (CPT) NCs were made (Fig. 5) utilizing the antisolvent precipitation approach in the work by J. Wang *et al.*^91^ These NCs boosted aqueous dispersion, increased stability, improved drug loading, and sustained circulation. The release of pH-sensitive drugs also reduces their toxicity toward normal cells. These NCs had efficient anticancer properties in serving CD44 overexpressed cancer cells. This occurs due to their precise distribution and enhanced uptake *via* CD44-mediated endocytosis. This unique drug formulation's physicochemical characteristics, *in vitro* anticancer performance, CD44 targeting possibilities, and associated acting mechanism have all been thoroughly assessed and investigated.^91^ HepG2, MCF7, and MDA-MB-231 cell lines were used. The HA coating served as a targeting ligand, hydrophilic layer, stabilizing agent, and protective coating. Therefore, this HA-based CPT formulation is anticipated to be a reliable and secure method of drug administration. Due to their targeted administration and improved absorption *via* CD44-mediated endocytosis, HA-coated CPT NCs demonstrate dramatically improved anticancer activity when treating cancer cells that overexpress CD44.^91^ In addition, they have better biocompatibility and antimigration properties. The molecular mechanisms linking this therapy to intrinsic mitochondria-mediated apoptosis may include a rise in the Bax to Bcl-2 ratio and the activation of P53. This could be the possible mechanism of anticancer activity with minimal adverse effects.^91^ The creation of platinum (Pt) NCs on biodegradable porous silicon nanotubes has been reported by N. T. Le *et al.*^92^ On investigating the anticancer abilities *in vitro* by promoting apoptosis, the immobilized Pt NCs caused high toxicity toward HeLa cells. Thus, they concluded that this may be a future cancer treatment approach.^92^ Y. M. Kim *et al.* developed cellulose-derived NCs to deliver polymeric siRNA to cancer cells.^93^ To create cationic NCs, hydrothermal desulfation and chemical modification were performed. Then, polymeric siRNA was created using a two-step method that included rolling circle transcription and Mg^2+^ chelation. NCs demonstrated excellent gene demolishing, upgraded enzymatic stability, and caused apoptosis in an *in vitro* investigation.^93^ Nanoparticles (NPs) were initially generated from a bioprecursor using goat blood and the RBC lysis method by S. Vellingiri *et al.* Later, it was coated with PEG and mixed with DOX to produce NCs.^94^ Both *in vitro* and *in vivo* experiments used the A549 lung cancer model. The study of the cell lines demonstrated intracellular iron recognition, morphological flaws, and dose-dependent cytotoxicity. Nuclear chemotherapy and cellular uptake may be carried out using these NCs, and DOX signals in the nucleus indicate this. In the histological investigation, DOX may be seen to suppress the development of cancer and have no adverse effects on healthy organs. As a result, NCs derived from natural sources can be employed as anticancer agents.^94^ In their work, J. Zhao *et al.* newly created PTX NCs using a bottom-up approach.^62^ These were redesigned with folic acid and PEG derivatives. The subsequent NCs had rod shapes with better drug loading. The *in vivo* investigation was performed on nude mice carrying 4T1 orthotopic breast cancer. The findings show that PTX inhibits tumor development and intratumor accumulation, making these NCs appealing for cancer-targeted treatment.^62^ NCs were hence used as anticancer drugs to provide better advancements in cancer therapy by following various pathways, leading to apoptosis or cell death.

### 3.3. Immunomodulation and immunotherapy through nanocrystals

Integrating nanotechnology with cancer immunotherapy has advantages, such as a targeted carrier system that can be finely adjusted for the necessary charge, surface alteration, size, and shape to enhance delivery effectiveness. In particular, magnetic navigation and the conjugation of targeting agents can improve the localization of the treatment to the target region.^95^ A liposome containing ursolic acid (UA) was created by N. Zhang *et al.* using hydroxypropyl beta cyclodextrin (HPβCD).^96^ The formation of crystalline structures in the liposomes in extended-release *vivo* investigations employing mouse tumor models results in the extended release of UA. With a considerable shift in regulatory T cells, these NCs were discovered to be nontoxic in 4T1 triple-negative breast cancer cells (TNBCs) and inhibited IL-10 and STAT5 phosphorylation secretion. When the UA-liposomes were given *in vivo*, they drastically changed the immune cells in the cancer microenvironment. To alter the immune system's antitumor activities for cancer treatment, this formulation may develop immunological factors.^96^ In addition, they can aid in preventing tumor development. Such a formulation may be quite popular in immunotherapy as well as in combination treatment with specific drugs.^96^

**Fig. 6:**
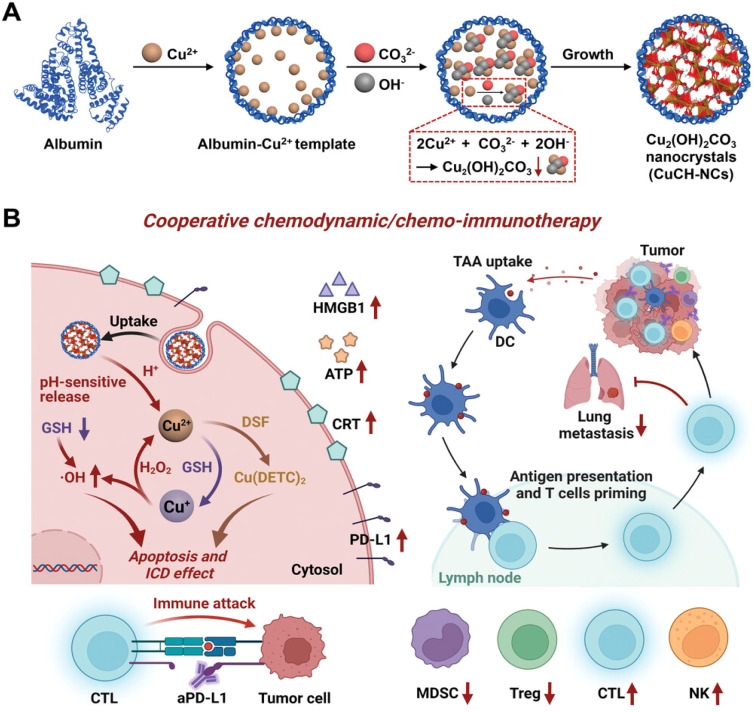
(A) Synthetic process of biomineralized NCs. (B) *In vivo* chemoimmunotherapy/chemodynamics of NCs in combination with DSF and aPD-L1. Reprinted with permission from ref. 97. Copyright 2022, *Advance Materials*.

In addition to immunotherapy targeting TNBCs (Fig. 6A), pH-activatable proenzymes have been reported by T. Li *et al.*^97^ Albumin nanocages were filled with copper(ii) carbonate hydroxide NCs (CuCH-NCs). For the synthesis and application of (CuCH-NCs), biomineralization took 120 min. TEM shows monodispersity with a 9.4 ± 0.4 nm diameter, whereas a hydrodynamic size of 18.8 nm with a narrow distribution was observed in DLS. 4T1 tumor-bearing mice were used for the *in vivo* investigation with 1 mg kg^−1^ dosing. 24 hour post-injection, the highest tumor accumulation was observed and eliminated through normal tissues.^97^ By these NCs, the pH-dependent release of Cu^2+^, which then undergoes glutathione (GSH)-mediated reduction to Cu^+^ for catalyzing the conversion of H_2_O_2_ into hydroxyl radicals (OH), leads to an increase in apoptosis and a suppression of cell growth. NCs demonstrate a synergistic effect with antiPD-L1 antibody because it is responsible for the elevation of PD-L1 and immunogenic cell death following combination with disulfiram (DSF). TNBCs have antitumor activity due to the activation of innate and adaptive immunity, which causes immunosuppression (Fig. 6B). These CuCH-NCs show tumor-selective anticancer effects with the synergized effect of chemotherapy with immunotherapy against TNBC. CuCH-NCs release Cu^2+^ in an acidic medium and simultaneously undergo GSH reduction to form Cu^+^, which acts similar to a Fenton reaction to produce chemodynamic activity. This further leads to H_2_O_2_ disintegration into its respective radicals, causing it to become a strong agent for CDT-mediated cell injury.^97^ Also, a combination of NCs with DSF displayed an upgradation in cellular damage and was favorable for chemotherapy. Hence, this formulation can be a safe and efficient approach by providing better tumor accumulation and penetration against aggressive cancers.^97^ In a different investigation by Z. Meng *et al.*, a bottom-up formulation of polydopamine (PDA)-coated insoluble supplementary immune NCs of imiquimod (R837) was prepared.^98^ To achieve immunomodulation, immune stimulation, and immunogenic cell-killing effects, these NCs were put into chitosan hydrogel. It was shown that the increase in cytotoxic T cells caused the tumor to transform into an *in situ* vaccine. This increases cancer immunity close to the tumor site. Therefore, it was forbidden for the cancer to develop, spread, or even recur.^98^ By combining iron oxide NCs with nitric oxide (NO) donors, Chen *et al.* created a pH-responsive nanosystem to treat pancreatic cancer.^99^ The development of stimuli-responsive nanosystems might be utilized to initiate an *in situ* catalytic cascade reaction and modify the antitumor immune system. As a result of the active microenvironment's production of NO, the tumor's blood supply expanded, allowing for orderly drug delivery. NCs produce oxygen–nitrogen species (RONS) and cytotoxicity through a NO reaction.^99^ Due to the catalytic cascade events regulated by the NCs, tumor-associated macrophages (TAMs) shifted to a proinflammatory M1 phenotype and tumor infiltration of effector T cells was promoted, resulting in efficient antitumor immunotherapy.^99^ By hierarchical bimetallic supra-nanostructures technique, snowflake-like Au NCs were prepared.^100^ A synergistic combination of radiotherapy and antiprogrammed death ligand 1 (aPD-L1) immunotherapy was achieved. Triggered radiation allows release (aPD-L1). The combination of primary Au and Ag NCs generates ROS by the radiation. The combined radiation treatment and NCs influenced immunogenic cell death (ICD) and the up-regulation of PD-L1.^100^ This treatment was found to increase the tumor-specific adaptive immune responses. They subdue the development of tumors by functionalizing cytotoxic T lymphocytes (CTLs) and lowering the T cells (Tregs). This release results in immunotherapy by a combination of radiation and drug present in NCs. A synergistic effect is observed in immune response with lower side effects.^100^ Similar research on immunotherapy was explained for primary and secondary tumors by altering the magnetic field.^101^ They observed the inhibition of cancer metastasis, and reoccurrence was also prohibited.^101^ In another study, pancreatic cancer was treated with magnetic manganese-zinc ferrite NCs. ICD and ferroptosis provided immunotherapy with minimal adverse effects.^102^ Arsenic(ii) sulphide NCs of size 93.14 ± 0.49 nm with PEG ligation were synthesized by gel method for breast cancer. The immune response triggers B cells because NCs lead to cancer cell death. They also included chemotherapy with immunotherapy for the treatment of breast cancer.^103^ Therefore, combining NCs with the immunity of the biological system cancer treatment based on immunomodulation, comprising immune suppression or immune stimulation therapy can be designed to treat tumor cells.

### 3.4. Biotherapeutics

Biotherapy plays a new role in cancer treatment. It is a form of treatment that reinforces the body against the adverse effects of other therapies and utilizes the body's immune system for protection against infection, cancer, and other disorders.^104^ Biotherapy includes probiotics,^105^ vaccine,^106^ cancer gene therapy,^107^ Antiangiogenesis therapy,^108^ other therapies involving immunomodulators, monoclonal antibodies, cell therapy, DNA, RNA, and siRNA have been used for trials and research.^109^ One such therapy is explained by Y. M. Kim *et al.*^93^ They synthesized cellulose NCs by surface modification. These NCs were designed to transport siRNA to tumor cells. Orderly hydrothermal desulfation processes were employed for this preparation. Chelation of Mg^2+^ and rolling circle transcription are two-stage processes responsible for the formation of NCs along with chemical changes. The complexation efficiency was altered to maximize drug loading and release in the cytoplasmic environment. *In vitro*, apoptosis was demonstrated by NCs, and gene breakdown and enzymatic stability improved. They concluded by presenting a treatment plan for cancer using RNAi.^93^

**Fig. 7:**
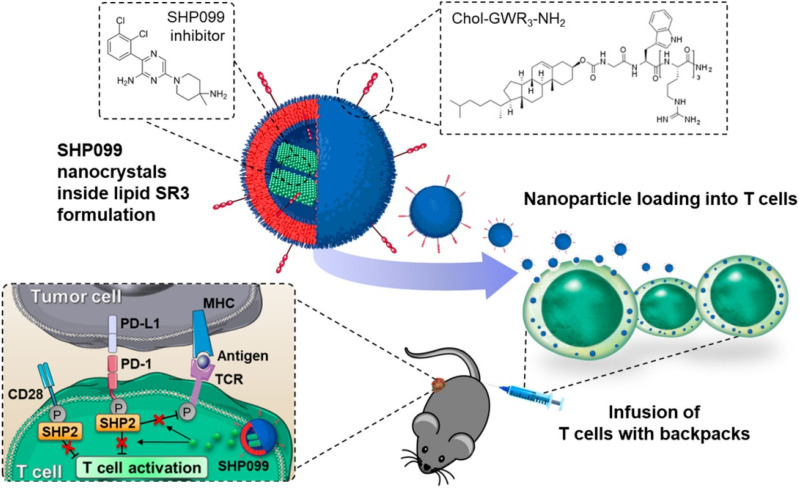
T cell therapy by loading of SHP2 inhibitor NCs. Reprinted with permission from ref. 110. Copyright 2022, *ACS Nano*.

In another study, they presented a technique to increase the effectiveness of T cells by loading them with SHP099, an SHP2 inhibitor.^110^ SHP099 formed nanocrystals inside the lipid vesicles mainly due to loading into lipid NPs covered with triarginine motifs (Fig. 7). This permitted high loading efficiency and sustained retention of SHP099 NCs into T cells. SHP099, which was cell-loaded, facilitated the long-lasting reduction of the PD-1/PD-L1 signaling and elevated T-cell activity.^110^*In vivo* study shows that tumor-homing T lymphocytes can travel with the cargo, boosting their tumor accumulation. By successfully blocking the PD-1/PD-L1 checkpoint signal, adoptively transferred SHP099 packed T cells in a solid tumor model provided total tumor eradication and persistent immunological memory against tumor rechallenging in all subjects treated in a solid tumor model.^110^ They show that combining T-cell treatment with SHP2 inhibition is an approach to therapy that has assurance. They also show that the lipid nanocrystal platform is an option for T cell loading of immune-regulating agents.^110^ Hence, biological factors can also lead to the form of therapy with the support of NCs to treat the cancer.

### 3.5. Radiotherapeutics

Radiotherapy involving high-energy electromagnetic radiation from X and Gamma rays is becoming popular in cancer treatment.^111^ Radiotheranostics combines targeted radionuclide treatment with molecular imaging (most often PET and SPECT), usually using radionuclides that generate α-, β- or auger-radiation. The ability of radio theranostics to target and eradicate cancer cells has led to their widespread use in oncology.^112^ The utilization of radio theranostics possesses several benefits, being able to assess the biodistribution of the targeted drug visually, the ability to select and monitor candidates for targeted therapies, possibly reducing the risk of nonresponse to treatment, and a decrease in systemic side-effects in comparison to nontargeted therapies.^113^ As an illustration, S. Imlimthan *et al.* produced cellulose NCs that contained vemurafenib.^114^ These were examined in the YUMM1.G1 mouse model of lung metastatic melanoma that carries the BRAF V600E mutation. Testing carried out *in vitro* reveals NCs exhibited release studies, cell growth inhibition, stable radiology, and cellular uptake. NCs are retained in the liver, lung, and spleen during *in vivo* biodistribution.^114^ Longer mouse survival, which demonstrated the benefits of combination treatment for improved results, was caused by the continuous release of vemurafenib through the beta particles released by 177Lu. The effectiveness of the theranostics, when given intravenously, is revealed by the potential of these NCs through the improved therapeutic action in the animal model.^114^ For tumor-specific radiotherapy L. Wang *et al.*^115^ created NCs from iridium. Intensified DNA damage and nucleus targeting was observed by these NCs in the cellular radiotherapeutic examination. NCs were modified with PEG and RGD. Facilitation of tumor cell membrane and nucleus targeting takes place due to peptides and trans-activators of transcription protein (TAT). X-ray radiation with these NCs causes DNA lesions and piles up tumor cells within the nucleus.^115^*In vivo* studies done in 4T1 tumors xenografts bearing nude mice evaluate demolishment of carcinoma. With photoacoustic imaging techniques and the photonic NIR adsorption of NCs, efficient tumor treatments have been developed.^115^ Similarly, L. Wang *et al.* created iridium NCs.^116^ For effective tumor targeting and localization, dual targeting by the nucleus with TAT proteins and tumor cell targeting RGD peptides were employed. They explained that this targeting influenced tumor therapy.^116^ Li *et al.* derived NCs of gadolinium oxide (Gd) through the polyol method to explain the radio-sensitizing functionality in nonsmall cell lung cancer (NSCLC) from cell lines exposed to carbon ions.^117^ The sensitizer effectiveness ratio at the 10% survival threshold was correlated with the total Gd concentration in NSCLC cells. NCs lead to cell cycle arrest and DNA damage due to the rise in reactive oxygen species (ROS) and hydroxyl radical secretion in radiated cells.^117^ Owing to carbon ion radiation, autography and apoptosis were found to be increased by these NCs. Hence, these NCs may act as prominent theranostics matter in carbon ion radiotherapy.^117^ Immunotherapy obtained with radiation is also an example of radiation therapy carried out by B. Choi *et al.*, as explained earlier in the above section.^100^ Therefore, irradiation with X-rays and gamma rays can help to detect and treat cancer in various forms.

### 3.6. Nanocrystals mediated photothermal therapy (PTT)

A NIR laser is employed in treatment to emit light to a particular location, which triggers the nanostructures gathered within the tumor.^118^ A localized hyperthermia effect occurs by transforming the NIR laser energy into heat, which NCs mediate.^119^ Hence NCs should have tumor specificity for better effect.^120^ The PTT mechanistic examinations revealed that when the tumor tissues are heated to a temperature of 41 °C, irreversible cell death can occur rapidly owing to vessel formation and degradation of the protein.^121^ High tumor penetration into biological tissue buried deep within requires PTT. Due to its smaller absorption and scattering in biological tissues and higher photon energy compared with ultraviolet light radiation, near-infrared (NIR) light is the primary laser for PTT applications. NIR light is primarily divided into two; the first NIR (700–950 nm) and the second NIR (1000–1700 nm) biological window.^122,123^ For example, PTX NCs were prepared by coating them with poly-tannic acid (pTA). Fe^3+^ plays a significant role in PTT while developing a coating over NCs.^124^ These NCs had a 470 nm size and were rod-shaped. PTA exhibited a photothermal effect when subjected to an 808 nm laser, and this action might be boosted with additional Fe^3+^. For many malignant cell lines, the cellular absorption of PNC-pTA was improved with the addition of pTA, as it exhibited outstanding photostability.^124^ These NCs may act as prominent theranostics matter in carbon ion radiotherapy. *In vivo*, results of NCs reveal hard effects on tumor suppression upon radiation exposure and lenient photothermal effect. They concluded that the NCs had greater therapeutic activity from combining photothermal and chemotherapy.^124^ In another study, iron oxide NCs were prepared using the ligand-assisted coprecipitation method.^125^ These NCs offered T2-weighted magnetic resonance (MR) imaging and photothermal ablation properties ideal for cancer theranostics. With the different quantities of ligand optimization that could be achieved, they have shown a 150 nm size of NCs. Without being exposed to radiation, HT-29 colorectal cancer cells did not show any cytotoxicity toward NCs.^125^ They also show greater photothermal conversion efficiency (21.2%), but cell viability was decreased due to radiation. The permeability of the membrane and secondary protein structure can be changed by apoptosis. *In vivo* studies, NCs with magnetic field (MF) usage greatly boosted tumor accumulation by four times.^125^ This resulted in a three times higher T2-weighted MR signal than that obtained from a commercial (Resovist®) and excellent photothermal efficacy (roughly 53 °C) for cancer treatment.^125^ By the high-temperature solid-phase method H. Li *et al.* synthesized rare earth ions-doped Ba_2_LaF_7_ NCs.^126^ Er^3+^ and Nd^3+^ emission bands are heavily isolated by optical temperature measurement according to fluorescence intensity ratio, which increases sensitivity. Relative sensitivity is raised due to an increase in temperature. NCs show a better photothermal effect with laser pumping power when compared to the actual temperature and fitting curve, hence used in imaging.^126^ They designed a multifunctional system which acts on living tissues to examine its optical assessment and photothermal conversion capability.^126^ Z. Li and L. Rong also explained the synergistic effect by combining therapy of PTT, starvation, and immunotherapy for cancer using R837.^127^ Here, both the primary tumor and its metastasis are suppressed, and a function similar to vaccination is provided due to a persistent immunological memory effect for tumor prohibition.^127^ PTT can hence help treat cancer by triggering and accumulating within the tumor cells. This can also be used in imaging and detecting the cancer cells and further combined with therapy to treat the cancer.

### 3.7. Photodynamic therapy (PDT) through nanocrystals

PDT, also called photo radiation therapy, does FDA approve the first combination,^128^ and it has long been used in anticancer therapy. Photosensitizer, light, and oxygen are essential components of this therapy.^129^ Minimum toxicity, controllability, and noninvasiveness are some of its advantages.^130^ By converting molecular oxygen to ROS, PDT ensures the inhibition of cancer cells.^131^ Hence hypoxic condition restricts the efficiency of PDT.^132^ Various examples of NCs using PDT have been created and evaluated.

**Fig. 8:**
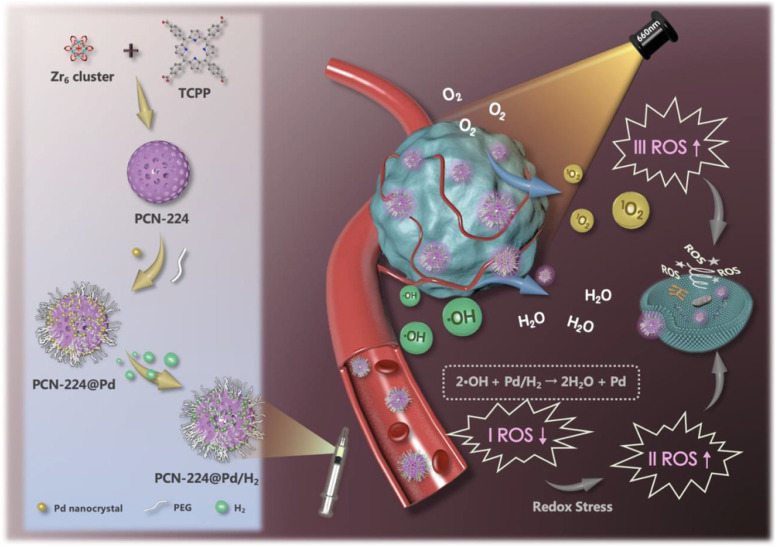
Synthesis of NCs for effective synergistic hydrogen/photodynamic therapy. Reprinted with permission from ref. 133. Copyright 2020, *Advance Functional Materials*.

A nanoscale porphyrinic metal–organic framework (NPMOF) with palladium NCs integration was developed.^133^ NCs function as hydrogen vehicles and are supported by the NPMOF, which acts as a photosensitizer for PDT. NCs were synthesized from the PCN-224 NPs with Zr_6_ cluster through the solvothermal method (Fig. 8). In the TEM characterization, 10 nm NCs were homogeneously spread over NPs and zeta potential from 21.9 to 10.7 mV. NCs with PEG ligation proved to be a better alternative for *in vivo* application by intravenous injection.^133^ The redox steady state of the cancer microenvironment was altered to adjust to the combined effect of cell death. Combining hydrogen treatment with PDT may substantially modify the TME's steady-state redox balance by ˙OH reduction and O_2_ excess production, accelerating redox stress-induced tumor cell death *via* various pathways.^133^ From this angle, it is anticipated that concurrent hydrogen/photodynamic treatment could improve tumor inhibition and increase therapeutic tolerability. To examine the reductivity and hydrogen release behavior, methylene blue was used as an oxidative probe without a Pt catalyst, as it will combine with NPs and help to detect the hydrogen. The hydrogen-affinitive Pd NCs and the porphyrin photosensitizers enable the Pd NCs with PCN-224 nanoparticles to achieve reductive hydrogen transport and light-activated O_2_ production. The hydrogen-containing nanosystem (PCN-224@Pd/H_2_) made a tumor-specific hydrogen treatment possible, displaying a prolonged reducing hydrogen release profile in the cell and solution.^133^ This resulted from the final hydrogen-containing nanosystems' constant hydrogen release and producing singlet oxygen. Therefore, both *in vitro* and *in vivo* assays demonstrated that the nanosystem possessed a high degree of biocompatibility and attained synergistic hydrogen/photodynamic therapy, which helped to enhance the anticancer effect in contrast to the single treatment.^133^ As a result, the synergistic combination of precision PDT and tumor-specific hydrogen therapy offers major potential for cancer treatment. Therefore, cancer-targeted therapy with PDT led to effective cancer treatment with fewer side effects.^133^ In another study, O_2_-producing NCs of catalase were used to resolve the hypoxic condition for the enhancement of PDT.^134^ Methylene blue (MB) was used as a photosensitizer, and different factors improved the efficiency of PDT. Firstly, O_2_ supply to hypoxic conditions occurs due to prolonged deposition of H_2_O_2_. This takes place due to the catalytic activity and stability of NCs. Singlet O_2_ is produced by the O_2_ and MB present within the NCs.^134^ MB is triggered to bear more singlet O_2_; as MB is uniformly distributed in the NCs lattice, cluster formation is also avoided. These factors raise PDT activity *in vitro* and *in vivo*. This development showcases new aspects of other O_2_-dependent therapies such as immunotherapy and radiotherapy.^134^ PTT and PDT were combined in one of the investigations. In a Schiff-base reaction, they generated cellulose NCs by altering the amido carbon dots (NCDs) and aldehyde.^135^ When creating a hydrogel, NCDs act as both phototherapy agents and crosslinkers. The NCDs demonstrated a high photothermal reaction with an efficiency of 77.6%, among the highest for photothermal agents, and a strong singlet quantum yield of 0.37 over a single 660 nm light-emitting diode illumination.^135^ Studies on nude mice carrying the B16F10 tumor were done *in vivo*. *In vitro* and *in vivo* tests reveal that the hydrogel has larger cancer suppression and is nontoxic. Thus, this depicts a more modern method for treating cancer.^135^ These applications explain the importance and need of NCs in theranostics, especially in cancer detection and treatment. Several other studies are still undergoing progress to improve and enhance their efficiency. Formulations are still struggling to reach further preclinical and clinical trials. Hence, more effective combinations and syntheses are required to upgrade the novel nanosystem.

## 4. Theranostics including imaging and therapy by nanocrystals

Nanodelivery complexes' multifunctional properties have drawn attention to the rise of theranostic (therapeutic and diagnostic) cancer-fighting strategies that unite a variety of pharmacological agents with imaging probes to track and examine the therapeutic agents circulated across the body.^136^ The real-time analysis of the proliferation or breakdown of cancer cells is given by theranostics.^137^ To recognize vascular inflammation by means of molecular imaging, NCs have been investigated as contrast agents. *In vivo* studies employing quantum dot as a contrast agent for molecular imaging has also shown excellent results. A preclinical investigation revealed the high sensitivity and specificity of molecular ultrasound imaging for disease recognition, categorization, and therapeutic response tracking. Microbubbles may be used to diagnose cancer with great potential.^138^ Some studies show the effective combination of such theranostics involving imaging and treatment.

**Fig. 9:**
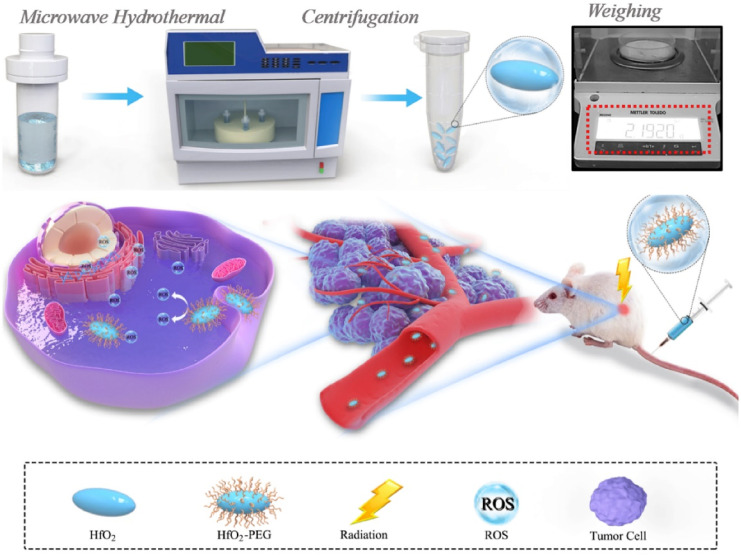
Synthesis process of HfO_2_ NCs and enhanced radiotherapy performance against the tumor. Reprinted with permission from ref. 67. Copyright 2020, *Biomaterials*.

For example, Y. Li *et al.* developed nanoassemblies (NAs) of HfO_2_ for intravenous injection through microwave-hydrothermal synthesis to enhance the effect of radiotherapy.^67^ NAs upon X-ray radiation killed the tumor cells by promoting free radical production. This led to an improvement in the responsive breast cancer cells (Fig. 9). NAs were ligated with PEG, which helps in CT imaging in a 4T1 breast tumor model. The inhibition of cancer cells was observed to be efficient through both intratumoral and intravenous administration.^67^ There has never been extensive research on how HfO_2_ NPs behave *in vivo* after intravenous injection, endangering pharmacokinetics, body distribution, and radiotherapy effectiveness. They also found the excellent biocompatibility of HfO_2,_ which can help in clinical trials and both *in vivo* and *in vitro*, which was investigated for the enhancement activity of NCs through radiotherapeutics.^67^ The studies showed that HfO_2_ NAs-mediated radio-sensitization effectiveness increased cancer cell-killing capacity and antitumor performance. The *in vivo* injections were carried out through interventional and intratumoral routes. The surface modification prolonged systemic circulation, which helped in tumor accumulation for better therapeutic activity.^67^ No toxicity after prolonged effect was achieved due to the effective degradation and elimination of the NAs. Hence, they effectively combined the nanosystem with radiosensitive therapy guided by CT imaging for better diagnosis and treatment.^67^ A porous magnetic nanosystem (Mn(0.25)–Fe_3_O_4_-III NPs) with a self-assembling technique was developed by J. Xu *et al.*^139^ for ferroptosis and chemotherapy with the benefit of MRI to enhance its effectiveness. Fenton reaction activity and greater drug loading were achieved due to the alteration in the porous structure of NCs. Fe and Mn in the nanosystem revealed combined catalytic activity and were found to be six times more than that of pure NCs.^139^ This made anticancer therapy possible, depending on ferroptosis. They could also decompose H_2_O_2_ in normal conditions but were found to metabolize under acidic pH, showing pH-dependent activity for creating COH to destroy cancer cells. *In vivo* investigations confirm the synergistic anticancer activity by ferroptosis and chemotherapy with minimum side effects by MRI scanning.^139^ By depleting glutathione (GSH), S. Tang *et al.*^140^ produced NCs of Prussian blue from the berlin green precursor. Both tumor cells and dendritic cells undergo this change. With no negative impact on healthy cells, these NCs promote tumor-specific 3D photoacoustic imaging and phototherapy of the tumor zone.^140^ The maturation of dendritic cells and immunogenic cell death can be produced by increased phototherapy with the depletion of NCs. Thus, they maintained cancer-specific photoacoustic imaging and efficient photoimmunotherapy.^140^ Similarly, M. Chang *et al.* explained dual-modal imaging platforms through photothermal and photoacoustic imaging for cancer detection and treatment.^141^ Here, Au is employed to trap light on the NCs of neodymium vanadate (NdVO_4_). NdVO_4_ nanorods and plasmonic Au NPs were employed to create NCs, and PVP was utilized for biocompatibility and stability. These lead to the production of ROS and increased the photothermal conversion by 32.15%. They endocytosed HeLa cells and induced phototoxicity.^141^ Tumor suppression and a NIR light-triggered anticancer impact were suggested by *in vivo* research. These NCs were excellent for detection and therapy based on light conversion into thermal form with radiation.^141^ 2D monodisperse lanthanum oxyiodide (LaOI) nanosheets were first reported by L. Xu *et al.* by altering the morphology.^142^ These had a thickness of 3 mm, and the shape could be changed by modifying the solvent concentration. Nanosheet behaves as a contrast agent in enhancing CT imaging.^142^ pH-dependent release led to intracellular transport and better biocompatibility by the anticancer drug doxorubicin (DOX). The drug also depicted a loading efficiency of 300 wt%. *In vitro* results show enhanced tumor cell destroying activity with lower dosing of combined DOX and nanosheet than pure DOX.^142^ A recent work performed hydrothermal synthesis to produce NCs of hydroxyapatite that were trivalently substituted with Eu and Bi ions. To treat lung cancer, A549 cell lines were used. The radiosensitization capability is provided by the Bi ion, while the Eu ions were used to liberate photoluminescence characteristics. l-buthionine sulfoximine (l-BSO) is applied to the NCs' surface to convey the radiation. By preventing antioxidant formation in the cells, which causes cell death, it aids in boosting radiosensitization.^143^ In another study, platinum(ii) sulphide nanodots (PtS-NDs) were produced for CT imaging following photothermal treatment. These nanodots ranged in size from 2.1 nm to 4.5 nm. The treatment of breast cancer was carried out using 4T1 tumor-bearing mice. They effectively absorbed infrared light, provided photothermal conversion in the presence of NIR laser radiation, and were photobleach-resistant. As a result, it induces *in vivo* hyperthermia and photoacoustic signals at the tumor location. They concluded that the albumin-templated biomimetic synthesis offered an innovative approach to generate theranostic PtS-NDs for prospective therapeutic uses.^144^

Similar newer nanosystems were developed by J. Cai *et al.* for efficient treatment involving imaging and therapy.^145^ Also, the galvanic exchange of Au NCs on CuS nanoplate was studied using CT imaging and photothermal therapy.^146^ In addition to describing several MRI-based cancer detection strategies, Siddique *et al.* also discussed multimodal imaging, image-guided treatment, and combination therapies. They described how imaging-guided treatment might enhance radiotherapy. For killing cancer cells, such as those in lung cancer, PAI-guided PTT was explained. For improved clinical results, combination therapy targets a variety of cancers. To get around drug resistance, they often produce drug synergy.^147,148^ J. Moore *et al.* used AI, stochastic models, and diagnostic models to increase and obtain the most effective anticancer therapy because these approaches serve in advancing research concerning nanotechnology. They mentioned how the design, size, and functioning were changed. Nanosensor versatility allows for multimodal imaging and aids in developing diagnostic and therapeutic imaging procedures.^149^ NCs functionalization in imaging and therapy is a vast area to research for future applications as it helps identify and treat tumor cells. More investigations and studies are required in this direction to explore the field of theranostics by NCs.

## 5. Detection and diagnosis

Recognizing cancer at the initial stage is still challenging. Diagnostic facilities at present are insufficient in detecting cancer cells.^150^ This leads to the need to develop new and advanced strategies for detecting and diagnosing tumor cells for treatment. Ongoing research shows various recent approaches toward diagnosing and detecting cancer cells. One such study includes the synthesis of an organic metal framework NCs from the rare-earth element dysprosium (Dy) for the very first time.^151^ They were combined with tetrakis(4-carboxyphenyl)porphyrin (TCPP), which explains 2D structures. PD-L1 production was lower in cancer cells with the prepared NCs from *in vivo* and *in vitro* investigations.^151^ Sonodynamic therapy (SDT) can also be used apart from immunotherapy by making Dy-TCPP as the TCPP ligand is susceptible to ultrasound. It can also be used in MRI. Hence, cancer treatment consisting of immunotherapy and SDT on an MRI-guided platform without any drugs or external checkpoints succeeded.^151^ Due to the increasing effects of AuNPs on CdS NCs, R. Heidari *et al.* created an electrochemiluminescence (ECL) immunosensor for the precise measurement of p^53^ protein.^152^ A sandwich model of antip^53^/p^53^/secondary antip^53^ was formed, through which AuNPs were launched in Cds NCs on carbon electrode. The ECL intensity was enhanced with the introduction of graphene oxide during the fabrication of the immunosensor and the addition of AuNPs on the surface of the electrode.^152^ The developed immunosensor's linear range was 20 and 1000 fg mL^−1^, with a predicted detection limit of 4 fg mL^−1^. Detecting p^53^ protein in human spikes validates the originality of the immunosensor for cancer therapy.^152^

**Fig. 10:**
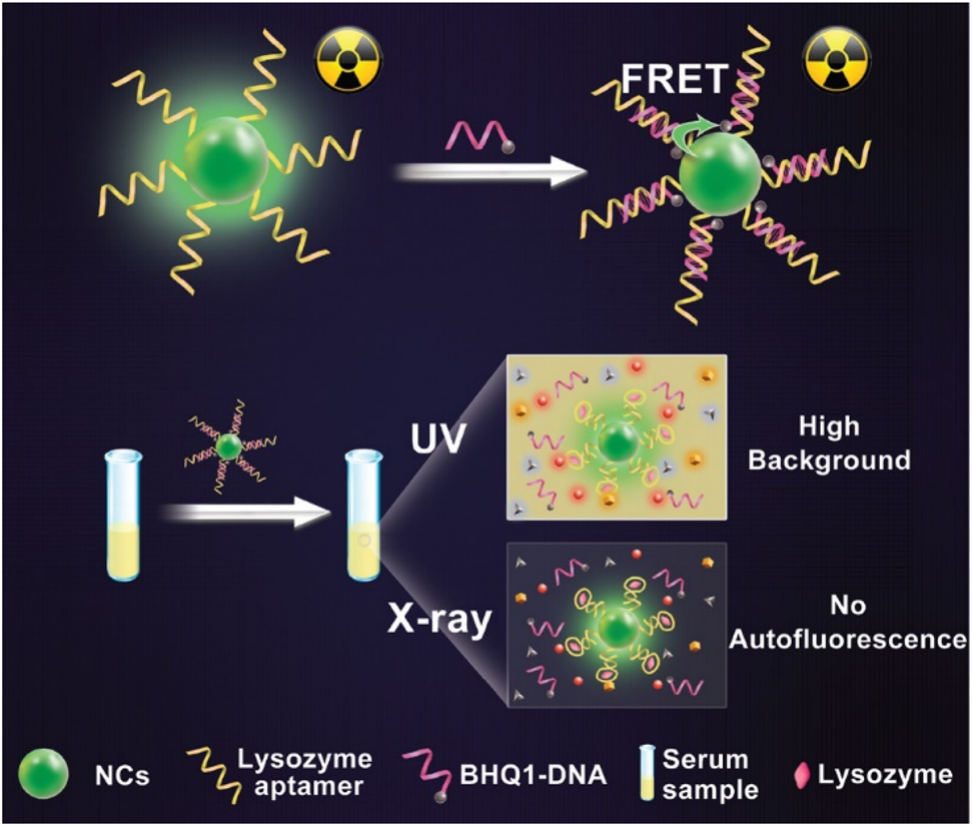
Autofluorescence-free detection of lysozyme using the NCs scintillator-based aptasensor. Reprinted with permission from ref. 147. Copyright 2019, *Analytical Chemistry*.

In another study, X-ray NCs based on oscillating aptasensor for detecting lysozyme biomarkers uniformly and sensitively through fluorescence resonance energy transfer (FRET) from human serum (NaLnF_4_: Tb@NaYF_4_)^153^ was used. The proposed NCs possess high sensitivity up to 0.94 nm and excellent accuracy due to the less X-ray dose as an energy source and high-efficiency illumination of heavy atoms-contained NCs (Fig. 10).^153^ The surface of NCs oscillators was modified using quencher-1-linked lysozyme DNA aptamers (BHQ_1_-DNA), in which BHQ_1_ can act as a nonfluorescent chromophore to block X-ray-induced luminescence. Characterization through TEM reveals the single crystalline structure of NCs. A remarkably high signal-to-noise ratio may be anticipated to find minimal lysozyme in complicated serum samples.^153^ Hence, they limited the generation of autofluorescence with greater sensitivity toward the detection of biomarkers for utilization in biomedicine.^153^ Similarly, Y. Zhao *et al.* developed electrode-altering bismuth sulfide NCs for identifying cytokeratin 18, a biomarker for bladder cancer. The detection time was less than 30 s.^154^ Also, the speed, simplicity, and harmless ability to identify the sensitive and specific electrochemical biosensor pointed to its potential utility in the early detection of bladder cancer and other disorders.^154^ As detection is an important parameter for diagnosing any disease, the advanced research undergoing for NCs might help in identifying an easier and more efficient source for imaging the tumor cells in the body.

## 6. Evaluation of nanocrystals by biophysical characterization

### 6.1. The effect of size on the nanocrystal formulation

The performance and properties of NCs can be heavily influenced by their size in several applications. The interference of size also hampers solubility, physical stability, therapeutic efficiency, and dissolution. It was observed that the surface energy is inversely proportional to the particle size of the NCs, leading to aggregation.^155^ Hence, optimizing and controlling the synthesis is important for the desired effect. The hardness is a crucial parameter for defining the resultant size of the NCs. For example, in the case of soft drugs such as PTX, the diameter of NCs is generally 300 nm.^156^ Factors such as stabilizer, solvent property, solvent antisolvent ratio, and drug concentration may affect the size.^156^ The pharmacokinetics of drug NCs also depend on the particle size; for example, the bottom-up and top-down methods employed to generate Riccardi D NCs yielded particles with mean sizes of 184 and 815 nm, respectively.^157^ The results demonstrate that the larger NCs improved drug exposure over 2.65 times, mean residence by 1.96 times, and maximum serum concentration by 5.61 times.^157^ Likewise, smaller oridonin NCs (103 nm) in a rabbit model exhibited a drug concentration–time curve equal to the drug solution.^158^ The larger crystals (897 nm), however, showed a markedly retarded elimination from the system.^158^ From these investigations, it is clear that larger NCs enhance bioavailability and sustain the elimination when compared to smaller structures even though they dissolve faster and minimize the phagocytosis by the reticuloendothelial system.^157,158^ Studies also suggest that the toxicity is dependent on the size of NCs. The toxicity profile rises with the increasing size of CeO_2_ NCs (30–50 nm), whereas 7 nm size was optimal for human cells.^159^

### 6.2. Activity of nanocrystals depending on the shape and surface charge

The fate of the NCs in biological systems is significantly influenced by their shape. The study results show that biodistribution, circulation time, cell absorption, and overall medication effectiveness in the body are greatly impacted by the NCs' proper form. Depending on the amount of mucus that may be penetrated, it is the first barrier for absorption.^160^ To investigate this parameter, Guo *et al.* prepared three different forms of NCs, namely, rod-shaped, flaky, and spherical.^161^ They observed that rod-shaped NCs have better transportability and enhanced permeation with efficient cellular uptake compared to other NCs in *in situ* intestine absorption.^161^ Also, in comparison to the spherical nanocrystals, the rod-shaped nanocrystals significantly increased cellular absorption, inhibited *in vitro* cell growth, and had a stronger anticancer effect *in vivo*.^162^

The surface charge (zeta potential) of a nanocrystal is a key feature that influences the self-assembly of nanoparticles, stability of the crystal, creation of nanoarchitecture, composition and performance of nanocomposites, chemical, and photocatalysis, as well as a wide range of applications.^163^ Another study explains that the minimum concentration required for ordered phase production rises with increasing surface charge, and the NCs become less viscous.^164^ Published articles suggested that negative zeta potential show negligible protein adsorption, whereas higher charge adsorbs more.^165^ Also, it was observed that in most cell lines, positive or neutral charge enters easily rather than a negative charge, which is usually employed in cancer cell lines. Hence, based on the surface charge, the localization of NCs to cytoplasm or lysosome is dependent.^166,167^

### 6.3. Bioavailability of nanocrystals based on their solubility and dissolution

NCs are usually formed with the drugs falling into the BCS class II, having low solubility but high permeability. They persist due to their lower solubility since dissolution and bioavailability are interdependent. Hence, it is difficult for water-insoluble or hydrophobic drugs to achieve bioavailability, leading to lower efficiency.^168^ 90% of pharmaceuticals are in the discovery process, and 40% treatments in the market are considered to have solubility issues. Quercetin, for instance, has an extremely low solubility in water (4 ± 0.3 μg mL^−1^). However, the NCs are more readily soluble (55–60 μg mL^−1^).^169^ The key characteristic of NCs is the dissolution or release, which can impact the *in vivo* safety, general bioavailability, and medication effectiveness. The drug particles' size and surface area majorly affect how quickly they disintegrate. The primary assessment indicator is the dissolution curve, which can highlight variations in product bioequivalence. It has been demonstrated that oridonin NCs dissolve more quickly than pure oridonin after 120 min.^170^ The dissolution comparison between powder form and NCs of the same drug investigation shows a higher rate value (73.74 ± 5.33)% for NCs and (6.10 ± 1.55)% for the powdered nintedanib (BIBF) drug in 10 min at pH 6.8. BIBF-NCs were absorbed in the gut by caveolae-mediated endocytosis, clathrin-mediated endocytosis, and micropinocytosis and had greater oral bioavailability (23.5%) with extended absorption time.^171^ Similarly, the plant compound cryptotanshinone (CTS) demonstrated antimetastatic activity *in vivo*. Its restricted use is due to weak oral bioavailability and water solubility. However, in a pharmacokinetic investigation on rats, W. Zhao *et al.* discovered that CTS NCs had an oral bioavailability 2.87 times greater than that of the pure drug.^172^

### 6.4. Impact of nanocrystal-induced cytotoxicity on biological systems

Toxicology testing is a major priority for drug registration because safety is the prime objective of pharmaceuticals. Cytotoxicity tests can provide the criteria for *in vivo* animal research, making them crucial for monitoring NCs. The cytotoxicity of oridonin NCs (ORI-NCs) on Madin–Darby canine kidney (MDCK) cells was examined *in vitro* by Sheng *et al.* Compared to free drugs, ORI-NCs substantially reduced cell viability at higher dosages (34, 84, and 135 g mL^−1^). Moreover, MDCK cells and ORI-NCs revealed higher endocytosis than the free drug.^170^ The anticancer effect of hyaluronic acid-coated (HA-coated) camptothecin (CPT) NCs has been demonstrated in another article by J. Wang *et al.*^91^ Various CPT formulations were checked to identify the NCs' cytotoxicity. The outcome of MTT indicates that MCF7 and MDA-MB-231 cells resist simple CPT. The survival of cancer cells was extensively decreased by CPT, proving that NCs had a better cytotoxic impact. HA-coated CPT NCs showed the strongest *in vitro* anticancer effectiveness, with IC_50_ values of 4.1–35.4 times less than free CPT and 2.2–23.9 times less than pure CPT NCs.^91^

### 6.5. Pharmacokinetic and pharmacodynamic changes due to nanocrystals

The pharmacokinetic (PK) profile explains the general behavior of drugs inside the body from absorption, distribution, metabolism, and elimination.^173^ Articles suggest that PK varies due to the variety of NC shapes.^161^ The investigated study results comment that flaky NCs have longer *T*_max_, leading to poor absorption in epithelial cells. However, longer *T*_1/2_ can be observed with spherical, flaky, and rod-shaped, resulting in prolonged circulation *in vivo*.^174^ Pharmacodynamics (PD) includes the binding of receptors, post-receptor effects, and chemical reactions and deals with the study of the biochemical, physiological, and molecular impact of drugs on the body. It can be affected by various factors. For the PD study of paclitaxel and niclosamide NCs in TNBC, D. Zhao *et al.* considered a mouse model with 20 days of observation by injecting an antitumoral injection.^80^ A study demonstrated the change in average body weight, tumor size, and intratumoral treatment residual. The combination administration group had the greatest success rate on the TNBC xenograft model after 20 days of therapy, obtaining a tumor suppression rate of 68.8%. The *in vivo* pharmacodynamic studies concluded the drug depot by injecting the NCs gel and prohibiting the TNBC. Thus, pharmacodynamics concludes what a drug does to the body when administered.^80^

### 6.6. Impact of nanocrystals on cellular uptake and stability in the system

One of the most significant procedures used to regulate molecular biological activity is cellular absorption, modulated by interactions between the molecule and the plasma membrane. An investigation by He *et al.* generated PTX NCs with FITC labels in three distinct sizes (500, 300, and 160 nm). A549 and Caco-2 cell lines were used to test the cellular absorption at 37 °C. Nystatin, a cell uptake inhibitor, can block the absorption of 160 nm particles in A549 cells showing caveolae-mediated uptake. On the other hand, the absorption of 300 nm PTX-NCs was energy-dependent caveolar endocytosis for nystatin and NaN^3+^ deoxyglucose. The uptake of PTX-NCs in Caco-2 cells that contained caveolae inhibitors on their surface produced similar results.^175^ Another study demonstrated how various nanomaterial coatings on the NC alter cellular uptake. After incubation, PTX-NCs coated with various surfactants or polymers, including FA-PEG-3400-NH_2_, folic acid (FA), Pluronic F-127, and Pluronic F-68, showed *in vitro* antitumor efficacy against KB-cells and a variety of cellular absorption mechanisms. The polydopamine (Dp) PTX-NCs displayed poor cellular absorption. Further, PEG coating decreased intracellular accumulation. The F-127-modified PTX-NCs had the least intracellular accumulation. However, the F-68-coated PTX-NCs demonstrated a boost in drug concentration. A minimal cellular absorption was seen in FA-PEG-Dp-NCs with Dp coating.^176^ NCs stability has two components: physical stability (sedimentation, crystalline state, crystal growth, and agglomeration) and chemical stability (degradation and spoiling). Therefore, surfactants and polymers are needed to stabilize the NCs. The choice of stabilizer can impact their performance *in vivo* and subsequent formulations.^177^ In an article, they employed a medium milling technique and investigated the nitrendipine NCs (NTD-NCs) stability at different temperatures. The size of the particles was unaltered after 30 days of storage at 25 ± 2 °C. A slight increase in size from 268 to 300 nm appeared after 20 days of preservation at 4 ± 2 °C. This may result from drug molecules that are freely recrystallized on the surface of larger crystals. Also, at 40 ± 2 °C, the size changed. This indicates that the NCs are fragile at elevated temperatures.^178^

## 7. Clinical and preclinical studies on nanocrystals

Most clinical trials are in phase I and II.^179^ Complications such as bioequivalence, chemistry manufacturing, and control dissolution of the NCs delay a formulation's clinical advancement.^180^ Microfluidic devices could be used to enhance the performance *in vivo* and hence help reduce the development of bioequivalence studies in clinical or human trials.^181^ Clinical trials may help in fixing the combination therapy. For example, paclitaxel and lapatinib perform the best when given in combination. Phase III studies on breast cancer revealed significant anticancer effects on collaborative administration. Inspired by this, they are given in combination. Even though they do not reach the target site simultaneously, their proportionate fraction can be effective because of their pharmacokinetic profiles.^182^ Disulfiram and copper–diethyldithiocarbamate (CuET) have been shown in numerous preclinical studies to efficiently kill CSCs of a variety of tumor models, including TNBC and hepatocellular carcinomas (HCC) by raising intracellular ROS and stimulating downstream pathways. This results in effective cancer therapy and nontoxic studies.^183^ However, poor anticancer activity was observed in clinical trials.^184–186^

CT-2103 poly(l-glutamic acid) compound is the most advanced polymeric paclitaxel combination and is being tested in clinical research. The parent chain of this compound has double carboxylic groups on it to increase its ability to transport the drug.^187^ Jarvis *et al.* also mentioned the drug product PanzemNCD (https://www.ClinicalTrials.gov Identifier: NCT00481455) in clinical trials with the drug 2-methoxyestradiol for ovarian cancer. This is administered orally and is currently in phase II.^180^ According to the official website of the clinical trials US government, panzemNCD is the only NCs-based formulation that went up for clinical trials and has been used for six studies to date. The colloidal dispersion of the drug panzem (Panzem®NCD) is in phase II interventional study (ME-CLN-004) in patients with ovarian cancer. The study started in October 2006 and was completed in November 2008. This study evaluated the efficacy, limited pharmacokinetics, and safety of 1000 mg Panzem®NCD administered orally four times daily to patients with recurrent or resistant epithelial ovarian cancer. In phase I and interventional studies (ME-CLN-002) from May 2006 to December 2009, the single center open-label study was done to evaluate the safety and efficacy of Panzem®NCD administered orally with a recombinant human monoclonal antibody against vascular endothelial growth factor Avastin (bevacizumab) administered intravenously in patients with locally advanced or metastatic carcinoid tumors. A single-center, open-label, phase 2 study (ME-CLN-005) was conducted to evaluate the antitumor activity as well as the safety and pharmacokinetics of Panzem®NCD administered in patients with recurrent glioblastoma multiforme (GBM) from January 2006 to December 2008. The open-label, multicenter, phase 2 trial (ME-CLN-006) to assess the antitumor activity, safety, and pharmacokinetics of Panzem® NCD in patients with metastatic, docetaxel refractory, androgen-independent prostate cancer was done from November 2006 to 2008. A single-center, open-label, phase II (ME-CLN-007), safety, and efficacy study of Panzem®NCD was administered orally in combination with protracted oral fixed-dose temozolomide to patients with recurrent glioblastoma multiforme from April 2007 to October 2008. Lastly, the phase II study (ME-CLN-008) of Panzem® NCD alone and combined with sunitinib malate in patients with metastatic renal cell carcinoma progressed from February 2007 to October 2009.

## 8. Challenges and future scope

Nanocrystals have extensively been used in medical applications for their physicochemical properties even though they could be toxic to the body and have adverse effects. Determining the nature of the NCs requires doing investigations on safety, effectiveness, and long-term destiny, such as *in vitro* and *in vivo*. The limitation of this nanotechnology is that only BCS class II medicines can be used in this method. In addition, the stability and production of NCs are influenced by the drug's molecular makeup. The preparation methods in laboratories involving top-down and bottom-up may vary from commercial manufacturing methods. Only a select few medication classes will be suitable for this strategy as an outcome. Production issues related to temperature, speed, homogenization, lyophilization, and centrifugation are among the other difficulties. Since yield might vary from one batch to batch, NCs must be synthesized and prepared with high repeatability. Hence, a preparatory phase during lab formulation should consider the scale-up manufacturing and identify the specific synthesis conditions related to temperature and pH as these may alter from place to place and improve the product yield. A slight change in other factors such as the type of solvent, crosslinker, stabilizer, amount of drug, the ratio of reactants, reaction time, and mixing conditions may result in greater biological or chemical undesirable outcomes with impurities. Hence, the synthesis of NCs should be optimized and reproducible. NCs with active targeting and tumor-specific uptake must be developed to increase the treatment efficacy and decrease nonspecific side effects. The permeability and retention effect can effectively enhance targeting. Various agents and specific components can be utilized to control nonspecific toxicity toward healthy cells, which leads to active targeting toward the tumor cells. Antiangiogenic peptides, antibodies, integrins, and mAbs are alternative methods used in active targeting. Researchers have also used RGD to target tumor cells specifically. Several other published reports suggest the targeting capabilities of NCs with other combinational effects. Thus, active targeting can enhance the anticancer effect with minimal side effects by nonspecific targeting. The diffusion and penetration of NCs into the cells are other important factors. Penetration across the endothelium at the tumor site and interstitial tissue, receptor-mediated entry to the target site, immune rejection (from spleen and liver), diffusion through the cytoplasm to release the drug, and entry inside the nucleus (if targeted) are the main parameters for intravascular delivery. Other routes such as mucosal membrane, skin, nose, lungs, and intestine may act as a barrier for diffusion resistance. Studies show that penetration and cellular uptake depend upon the sedimentation velocity and diffusion of NCs. They are autonomic in their size, shape, concentration, and morphology. The increase in sedimentation rate is thus directly proportional to the enhancement of cellular uptake. Another concern for the therapy is the elimination and biodegradability of NCs. It was observed that the liposomes and polymer-based NCs are biodegradable and get cleared easily from the body. On the other hand, metal-based NCs show slow metabolic degradation and are removed through urine and feces. Hence, the deposition of metal in the body may create toxicity. Studies propose that kinetics, fate, transport, distribution, clearance, and molecular effects can be identified with the morphology, shape, and size of the NCs. Also, it suggests the complexity *in vivo*, creating a major interest-gaining topic in research. Toxicology is an important parameter that should be considered before any clinical implementation. Inorganic NCs from metal sources such as Pt, Ag, ZnO, and CuO have a high risk of toxicity due to metal ions *in vivo*. Absorption, distribution, and excretion investigation shows the toxicity profile in animal model studies. The accumulation of drugs from the NCs leads to an increase in toxicity. Combined drug therapies may enhance recessive toxicity. Hence, the combination must be clinically approved before formulation and administration. Severe toxicity might result in long-term side effects, leading to organ damage. Due to the diversified properties of NCs, a specific character with tolerance is quite tough to detect. Thus, it is a basic need to significantly evaluate the toxicity profiles through various parameters, including genotoxicity, pharmacokinetics, pharmacodynamics, serum/blood/plasma, immunological responses, and tissue histopathology. Future demands and responsibilities include developing and investigating NCs that precisely target tumors. The goal should be to create a biocompatible and biodegradable formulation with minimal adverse reactions. In addition, the optimal delivery method and dosage should be investigated. Combinational methods can be beneficial in locating and destroying cancer cells. Much research is being conducted to create possible cancer theranostics. NCs approach can hence be an effective alternative technology in the near future. Applying the NCs approach could be convenient and helpful as a promising technique toward cancer research.

## 9. Conclusion

In research, nanoparticles and nanocrystals have been used for a few decades. NCs are renowned for their capacity to effectively increase drug permeability and bioavailability, particularly for BCS class-II medicines with low solubility. In addition, for more prolonged drug release from this novel drug delivery system, NCs with organic, inorganic, or hybrid origins can be made *via* top-down, bottom-up, or a combination of the two techniques. Investigations on cancer through NCs are still undergoing as they have the potential to expand the boundaries in cancer therapy. This review highlights the applications of NCs in cancer theranostics, including screening for the detection and medication of treatment. NCs use methods such as MRI, CT, luminescence, and biosensing to detect and identify the type and extent of cancer. MRI and CT scans employ X-ray radiation to locate the tumor cells. Electrodes, probes, and ligands can be utilized for biosensing with biosensors and biomarkers. Several cancer treatments have been developed. Immunotherapy is the process of boosting or inhibiting the immune system while harnessing immune cells such as B cells and T cells, to affect the immune system's response to particular cells. According to the findings, biotherapy has a new dimension that might be further investigated. It encompasses therapies using DNA, SiRNA, monoclonal antibodies, gene therapy, cell therapy, vaccines, antiangiogenesis therapy, and immunomodulatory bioactive substances. This treatment protects the body against illness, cancer, and infection. Imaging is receiving more interest in cancer therapy because it may be used to treat tumor cells with radiation using X-rays and gamma rays. Consequently, radiotherapeutics is regarded as a key technique for treatment and diagnosis. Targeting cancer cells gathered in the triggered zone by light from a laser source is called photothermal therapy. On the other hand, photodynamic therapy also uses oxygen and a photosensitizer. By converting molecular oxygen, it generates ROS to suppress cancer cells. Both cancer diagnosis and therapy are possible using theranostics. This provides effective diagnosis with successive treatment. Moreover, several articles have reported on synergistic therapies, including combinations of therapies. As a result, the therapy is improved because of the additive and synergistic effects. The biodistribution, delivery, accumulation, dynamics, kinetics, elimination, subsequent molecular effects, and the fate of NCs in a living organism can all be influenced by the shape, size, and morphology, which is more complicated in *in vivo* systems. As a result, this is an emerging field of research.

## Author contributions

DY, SM has conceptualized the manuscript. DY, MN, and SM has written the draft. SM has corrected and supervised the work.

## Conflicts of interest

There are no conflicts to declare.

## Supplementary Material
